# Definitive 3D-CRT for clinically localized prostate cancer: modifications of the clinical target volume following a prostate MRI and the clinical benefits

**DOI:** 10.1186/s40064-015-1138-9

**Published:** 2015-07-15

**Authors:** Shinsaku Yamaguchi, Takayuki Ohguri, Masami Fujii, Katsuya Yahara, Yoshiko Hayashida, Naohiro Fujimoto, Yukunori Korogi

**Affiliations:** Department of Radiology, Kitakyushu General Hospital, Kitakyushu, Japan; Department of Radiology, University of Occupational and Environmental Health, 1-1 Iseigaoka, Yahatanishi-ku, Kitakyushu, 807-8555 Japan; Department of Urology, University of Occupational and Environmental Health, Kitakyushu, Japan

**Keywords:** Prostate cancer, Magnetic resonance imaging, Clinical target volume, Tumor staging, Three-dimensional conformal radiotherapy

## Abstract

**Purpose:**

To evaluate the modifications of the tumor stage and clinical target volume following a prostate magnetic resonance imaging (MRI) to evaluate the tumor (T) staging, and the clinical benefits for prostate cancer.

**Methods:**

A total of 410 patients with newly diagnosed and clinically localized prostate cancer were retrospectively analyzed. The patients were treated with definitive three-dimensional conformal radiotherapy (3D-CRT). In all of the patients, digital rectal examination, transrectal ultrasound, prostate biopsy and computed tomography were performed to evaluate the clinical stage. Of the 410 patients, 189 patients had undergone a prostate MRI study to evaluate the T staging, and 221 patients had not.

**Results:**

Modification of the T stage after the prostate MRI was seen in 39 (25%) of the 157 evaluable patients, and a modification of the risk group was made in 14 (9%) patients. Eventually, a modification of the CTV in 3D-CRT planning was made in 13 (8%) patients, and 10 of these had extracapsular disease. Most of the other modifications of the T staging were associated with intracapsular lesions of prostate cancer which did not change the CTV. There were no significant differences in the biological relapse-free survival between the patients with and without a prostate MRI study.

**Conclusions:**

Modification of the CTV were recognized in only 8% of the patients, most of whom had extracapsular disease, although that of the T stage was seen in approximately one-quarter of the patients. Prostate MRI should only be selected for patients with a high probability of extracapsular involvement.

## Background

The standard imaging modality to evaluate the tumor (T) stage of the prostate cancer is currently transrectal ultrasound (TRUS). Recently, the use of magnetic resonance imaging (MRI) has been recommended for evaluating the T staging in patients with prostate cancer due to its high resolution (Turkbey et al. 2009; Barentsz et al. 2012; Kurhanewicz et al. 2008). However, the sensitivity and specificity of local staging with MRI vary considerably with technique and population; with rates ranging from 14 to 100% and 67 to 100%, respectively.

The T staging of prostate cancer plays an important role in planning radiotherapy (RT), as well as for predicting the prognosis. For example, in patients with T2 or better status in the low risk group, the clinical target volume (CTV) in RT planning should include the entire prostate only, while in patients with T3 or higher stage disease, the CTV should include the prostate, extracapsular disease and seminal vesicles. Patients with T3 or higher disease are more than six times more likely to have biochemical recurrence after radical prostatectomy compared to patients with T2 or better disease (Eggener et al. 2007).

However, there are a few clinical studies have investigated whether the addition of prostate MRI for T staging changes the CTV and/or improves the clinical outcomes (Chang et al. 2014; Mullerad et al. 2004). The purpose of this study was to assess the modifications of the T staging and CTV following a prostate MRI to evaluate the T stage, and the clinical benefits in patients with newly diagnosed and clinically localized prostate cancer treated with definitive 3D-CRT.

## Methods

### Patients

From January 1998 to December 2009, 410 consecutive patients with primary prostate cancer were included in this retrospective study with the following inclusion criteria: a pathologically confirmed adenocarcinoma of the prostate, treatment using definitive 3D-CRT with a total dose of 66 Gy or more, and neither nodal nor distant metastatic disease. The following patients were excluded: those who had hormone-refractory prostate cancer and those who underwent irradiation for the whole pelvic region. One hundred and eighty-nine of the 410 patients had undergone a prostate MRI study to evaluate the T staging, while the remaining 221 patients had not. The modifications of the tumor stage and CTV were evaluated in 157 (83%) of the 189 patients who underwent the prostate MRI study, because the image reading reports of MRI, which were written by diagnostic radiologists, were not available in the remaining 32 patients at the time of the evaluation. Written informed consent for treatment was obtained from all patients.

The pretreatment evaluation included a complete history, physical examination [including digital rectal examination (DRE)], prostate biopsy with histological evaluation, laboratory studies [complete blood count, creatinine, alkaline phosphatase and initial prostate-specific antigen (iPSA)]. Table 1 shows the patients’ clinical characteristics and treatments according to the use of a prostate MRI study. The risk groups were classified according to the guidelines of the National Comprehensive Cancer Network (NCCN) (2014) tumor stage. All patients had pathologically confirmed prostate adenocarcinoma, and 401 of 410 patients (98%) received neoadjuvant androgen deprivation therapy (ADT) (median 8.2 months; range 2.1–60.3 months); adjuvant ADT was continued in 128 (32%) of the 401 patients after the completion of RT (median 21.4 months; range 2.2–41.0 months). The median total duration of neoadjuvant plus adjuvant ADT was 8.5 months in 401 patients (range 2.1–129.0 months).

**Table 1:** The patient characteristics and treatment methods

	Prostate MRI		*p*
	Yes (n = 189)	No (n = 221)	
	No. of patients (%)	No. of patients (%)	
Age, years			0.50
Median (range)	73 (57–85)	74 (47–84)	
T stage^a^			0.82
T1–2	144 (76)	171 (77)	
T3–4	45 (24)	50 (23)	
iPSA (ng/mL)			0.91
<10	60 (32)	74 (33)	
10–20	57 (30)	68 (31)	
>20	71 (38)	79 (36)	
Not specified	1 (0)	0 (0)	
Gleason score			0.68
2–6	77 (41)	83 (38)	
7	65 (34)	82 (37)	
8–10	40 (21)	53 (24)	
Not specified	7 (4)	3 (1)	
Risk groups			0.48
Low	24	36	
Intermediate	60 (32)	72	
High	85	97	
Very high	16	15	
Not specified	4	1	
Date of treatment			<0.001
1998–2005	117 (62)	82 (37)	
2006–2009	72 (38)	139 (63)	
ADT			<0.001
<6 months	17 (9)	46 (21)	
≥6 months	171 (90)	167 (76)	
None	1 (1)	8 (3)	
Radiation dose			<0.0001
66 Gy	87 (46)	58 (26)	
70 Gy	100 (53)	162 (74)	
Other	2 (1)	1 (0)	
Hyperthermia			0.53
Yes	62 (33)	79 (36)	
No	127 (67)	142 (64)	

*MRI* magnetic resonance imaging, *iPSA* initial prostate-specific antigen, *ADT* androgen deprivation therapy.

^a^T stage after prostate MRI.

### Prostate MRI and interpretation

Routine prostate imaging for a work up in patients with newly diagnosed prostate carcinoma included TRUS and computed tomography (CT). In all 410 patients investigated in this study, TRUS and CT was performed by attending urologists, and the prostate MRI had been additionally selected to evaluate the T stage for some of the patients based on the decision of each individual attending urologist. Therefore, although the present study was retrospective and non-randomized in nature, the subjects included patients with and without a prostate MRI. Of the study population of 410 patients, 189 patients (46%) had also undergone a prostate MRI study at the time of the initial diagnosis and evaluation of the prostate cancer, and 221 patients (54%) had not undergone a prostate MRI study.

Because prostate MRI was performed after DRE, TRUS and CT, modifications, including up- or downstaging of the T stage and risk group after the prostate MRI studies, were retrospectively evaluated based on the subjects’ medical records, including image reading reports of MRI in each case. The image reading reports for prostate MRI were written by diagnostic radiologists for each patient.

As described below, the CTV was determined according to the risk group. Therefore, changes in the targets added at upstaging or deleted at downstaging in the CTV values obtained before prostate MRI, as appropriate for the risk group before prostate MRI, and after prostate MRI, as actually performed in the patients, were evaluated.

Scans were performed on a 1.5-Tesla MRI scanner using a phased array coil. Endorectal coils were not used in this study. The entire prostate gland and seminal vesicles were covered by axial T1-weighted spin-echo imaging (T1WI; repetition time/echo time, 450–550/8.4–8.9 ms, echo train length = 3, and the acquisition time was 2 min and 21 s) and axial T2-weighed turbo-spin-echo imaging (T2WI; 3,500–3,800/85–105 ms, echo train length = 17–18, and the acquisition time was 2 min and 34 s). These conventional images were obtained with a 4–5 mm slice thickness, 1.0 mm interslice gap, 200–250 mm field of view and a matrix size of 288–256 × 256–192. In 127 (67%) of the patients who underwent a prostate MRI study, gadolinium-enhanced imaging was performed. In 104 (82%) of these 127 patients, dynamic contrast-enhanced images were obtained using a 3D gradient echo sequence (LAVA; GE Healthcare, Milwaukee, WI, USA) with ultrafast image reconstruction by parallel imaging algorithms (ASSET factor, 2) in the axial plane (TR/TE 4.4–4.6/2.1 ms; flip angle, 12°, slice thickness, 3–5 mm; 320–400 mm field of view, matrix size of 320 × 192–256, and the acquisition time was 26 s).

### Radiation therapy

Radiation treatment was delivered to all patients with definitive intent. All patients were treated with a 10-MV linear accelerator using three-dimensional conformal RT (3D-CRT) planning. Computed tomography-assisted 3D-CRT planning (FOCUS or Xio; CMS Japan, Tokyo, Japan) was used to determine the radiation fields in all patients. All patients were treated in the supine position and underwent a planning CT scan.

A clinical target volume (CTV) was determined according to the risk group as follows: low risk, CTV1 was the entire prostate alone, and CTV2 was the same as CTV1; intermediate risk, CTV1 was the entire prostate plus proximal seminal vesicles, and CTV2 was the entire prostate; high risk, CTV1 was the entire prostate, gross extracapsular disease plus proximal seminal vesicles, and CTV2 was the entire prostate plus gross extracapsular disease and very high risk, CTV1 was the entire prostate, gross extracapsular disease plus the entire seminal vesicles, and CTV2 was the entire prostate and gross extracapsular disease. CT-MRI image fusion was not applied for the 3D-CRT planning, although prostate MRI was used to evaluate the T staging. Contouring of gross extracapsular disease was referred based on the prostate MRI.

The total planning dose was 66 Gy (n = 145), 70 Gy (n = 262), 72 Gy (n = 1), 74 Gy (n = 1) or 76 Gy (n = 1), and the fraction was 2.0 Gy once a day (five times/week) (**1**). The planning target volume (PTV) was delineated by contouring a CTV1 with a PTV1 margin of 1.2–1.7 cm during the initial 46 Gy in 23 fractions, and CTV2 with a PTV2 margin of 0.7–1.2 cm during the subsequent 20–30 Gy in 10–15 fractions. The beams were shaped using a multileaf collimator. All patients were treated with 3D conformational or seven-field conformal radiation with an isocentric technique.

One hundred and forty-one (34%) of the 410 patients were also treated with pelvic regional hyperthermia during 3D-CRT to improve the antitumor effects of RT. Hyperthermia was applied after irradiation once a week for radio-sensitization. An 8-MHz radiofrequency (RF)-capacitive regional hyperthermia system (Thermotron RF-8; Yamamoto Vinita, Osaka, Japan) was used (Abe et al. 1986).

### Follow-up

The length of follow-up was calculated from the date of the start of RT. The patients were followed-up at 1 to 3-month intervals during the first year, and at 3 to 6-month intervals thereafter. PSA measurement and assessments of the gastrointestinal and genitourinary morbidity were performed at each follow-up visit. Biochemical failure was defined according to the Phoenix definition (Roach et al. 2006). The clinical relapse-free survival (cRFS) was defined as the rate of freedom from local failure, regional failure and distant metastasis. The presence of bone metastasis was confirmed by bone scintigraphy, computed tomography or MR imaging. Soft tissue metastasis was confirmed by computed tomography or MR imaging.

### Statistical analysis

The Chi square tests and Mann–Whitney U tests were used to assess the baseline imbalances between the patients with and without prostate MRI. The Kaplan–Meier method was used to calculate the outcomes for the biochemical relapse-free survival (bRFS) and cRFS. The time period was calculated as beginning at the start of definitive 3D-CRT. To identify prognostic factors for the bRFS, univariate analyses were performed. The log-rank test was used for statistical comparisons between groups. Multivariate analyses using the Cox proportional-hazards model were also performed to determine the bRFS. Fisher’s exact tests were performed to evaluate the differences in the patterns of first failure between the patients with and without prostate MRI.

## Results

Table 2 shows the results for the modifications of the T stage, risk group and CTV in the 3D-CRT planning following prostate MRI study. Upstaging of the T stage was identified in 33 (21%) of the 157 patients, while downstaging was observed in six (8%) patients. Meanwhile, upstaging of the risk group after prostate MRI occurred in 10 patients, whereas downstaging was noted in four patients. Modifications of the CTV values were made in 11 patients with upstaging and two patients with downstaging after prostate MRI. Changes in the targets for the CTV, as added at upstaging or deleted at downstaging, are listed in Table 2. Table 3 shows the relationships among the risk groups classified according to the Prostate Cancer Risk Stratification (ProCaRS) risk group before prostate MRI, the upstaging of the T stage or NCCN risk group after prostate MRI, and the changes in the CTV after prostate MRI (Rodrigues et al. 2013). Eight (73%) of 11 patients with modifications of the CTV after prostate MRI who demonstrated upstaging were associated with the ProCaRS High-intermediate risk, High-risk or Extreme-risk groups before prostate MRI.

**Table 2:** A **s**ummary of the modifications for the T stage, risk group and CTV following prostate MRI studies in 157 evaluable patients

	No. of patients (%)	No. of patients with modifications of the risk group after prostate MRI (%) (change of risk)	No. of patients with modifications of the CTV in 3D-CRT planning based on the prostate MRI findings (targets added at upstaging or deleted at downstaging)
No change	118 (75)	–	–
Upstaging	33 (21)	10 (6)	11 (7)
T1c to T2a	10	0	0
T1c to T2b	10	1 (L → IM)	1 (pSV in CTV1)
T1c to T2c	3	2 (L → IM)	2 (pSV in CTV1)
T1c to T3a	2	1 (L → H)	1 (pSV and GED in CTV1, GED in CTV2)
T2a to T3a	1	0	1 (pSV and GED in CTV1, GED in CTV2)
T2a to T3b	2	2 (H → VH)	2 (eSV and GED in CTV1, GED in CTV2)
T2b to T2c	1	0	0
T2b to T3a	1	1 (IM → H)	1 (GED in CTV1, CED in CTV2)
T2b to T3b	1	1 (H → VH)	1 (eSV and GED in CTV1, GED in CTV2)
T3a to T4	2	2 (H → VH)	2 (eSV and GED in CTV1, GED in CTV2)
Downstaging	6 (4)	4 (3)	2 (1)
T3a to T2a	1	1 (H → IM)	0^a^
T3a to T2b	2	1 (H → IM)	0^a^
T3a to T2c	1	0	0
T3b to T2c	1	1 (VH → H)	1^a^ (eSV in CTV1)
T4 to T2a	1	1 (VH → H)	1^a^ (eSV in CTV1)

*L* low risk, *IM* intermediate risk, *H* high risk, *VH* very high risk, *pSV* proximal seminal vesicles, *eSV* entire seminal vesicles, *GED* gross extracapsular disease, *CTV* clinical target volume.

^a^A contour of the GED could not be depicted, because the GED confirmed or suspected in TRUS was not recognized in the prostate MRI study or by CT.

**Table 3:** Upstaging and changes in the CTV among the risk groups after prostate MRI according to the Prostate Cancer Risk Stratification (ProCaRS) risk stratification system

ProCaRS 6 categories^a^ before prostate MRI	Upstaging of the T stage after prostate MRI (n = 33) (%)	Upstaging of the NCCN risk group after prostate MRI (n = 10) (%)	Modifications of the CTV in 3D-CRT planning based on the prostate MRI findings (n = 11) (%)
Very low-risk	1 (3)	1 (10)	1 (9)
Low-risk	3 (9)	1 (10)	1 (9)
Low intermediate-risk	8 (24)	1 (10)	1 (9)
High intermediate-risk	7 (21)	2 (20)	2 (18)
High-risk	4 (12)	3 (30)	4 (36)
Extreme-risk	10 (30)	2 (20)	2 (18)

Very low-risk: T1–T2a AND PSA ≦6 ng/ml AND Gleason score ≦6. Low-risk: T1–T2a AND PSA >6 AND PSA ≦10 ng/ml AND Gleason score ≦6. Low intermediate-risk: T1–T2 AND PSA ≦20 mg/ml AND [PSA ≦10 ng/ml OR (PSA > 10 ng/ml AND {T1–T2a OR Gleason ≦6})]. High intermediate-risk: T1–T2 AND PSA ≦20 mg/ml AND [PSA >10 ng/ml AND (T2b/c OR Gleason 7)]. High-risk: [T3–T4 OR (PSA >20 ng/ml AND PSA <30 ng/ml) OR Gleason 8–10] AND % cores <87.5%. Extreme-risk: [(T3–T4 OR Gleason 8–10 OR PSA >20 ng/ml)] AND (PSA ≧30 ng/ml OR % cores ≧87.5%).

^a^Rodrigues et al. (2013).

**Figure 1:**
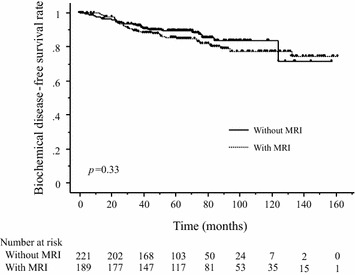
The 5-year biochemical relapse-free survival rates between the patients with and without a prostate MRI were 86 and 90%, respectively; there were no significant differences.

The median follow-up in all patients was 67.3 months (range 0.1–161.6 months), while that in the 189 patients with a prostate MRI study was 79.1 months (range 0.1–161.6 months), and that in the 221 patients without a prostate MRI study was 68.8 months (range 0.4–157.8 months). Table 4 shows the prognostic factors in the univariate analyses for the bRFS. The 5-year and the 3-year bRFS were 86 and 89% in the patients with a prostate MRI study and 90 and 92% in the patients without a prostate MRI study, respectively, and these values were not significantly different (Figure 1). The 5-year and 3-year cRFS were 96 and 98% in the patients with a prostate MRI study, and 97 and 99% in the patients without a prostate MRI study, respectively; there was also no significant difference in these values. In the multivariate analyses for the bRFS, the Gleason score was found to be a statistically significant factor, while the addition of the prostate MRI was not a predictive factor (Table 4). In the univariate subset analyses, among 315 patients with T1–2 disease, a prostate MRI study was not a significant predictor of the bRFS. It was also not a significant predictor among the 95 patients with T3–4 disease.

**Table 4:** Results of the univariate and multivariate analyses of factors associated with the bRFS after definitive radiotherapy

	n	Univariate		Multivariate^f^	
		5-year (%)	*p*	OR (95% CI)	*p*
Age (years)			<0.01	1.71 (0.99–2.99)	0.06
<70	130	82			
>71	280	90			
T stage^a^			<0.01	1.88 (0.96–3.71)	0.07
T1–2	315	92			
T3–4	95	74			
iPSA (ng/mL)^b^			<0.01	1.76 (0.83–3.72)	0.14
≦20	258	91			
>20	151	82			
Gleason score^c^			<0.01	3.06 (1.60–5.81)	<0.001
2–7	308	91			
8–10	92	75			
Risk group^d^			<0.01	1.30 (0.47–3.63)	0.62
Low-intermediate risk	191	92			
High-very high risk	214	83			
Date of treatment			0.11	1.41 (0.66–3.02)	0.37
1998–2005	199	90			
2006–2009	211	86			
ADT (months)^e^			0.45	1.07 (0.47–2.44)	0.87
<6	63	91			
≧6	338	88			
Radiation dose			0.06	1.51 (0.67–3.40)	0.32
<70 Gy	145	85			
≧70 Gy	265	92			
Hyperthermia			0.18	1.21 (0.65–2.26)	0.54
Yes	141	86			
No	269	88			
Prostate MRI study			0.33	1.57 (0.89–2.77)	0.12
Yes	189	86			
No	221	90			

*bRFS* biochemical relapse-free survival, *EBRT* external beam radiation therapy, *MRI* magnetic resonance imaging, *OR* odds ratio, *CI* confidence interval.

^a^T stage after prostate MRI.

^b^Excluding 1 patient with unknown iPSA.

^c^Excluding 10 patients with unknown Gleason scores.

^d^Excluding 5 patients with an unknown risk group status.

^e^Excluding 9 patients without ADT.

^f^The 388 evaluable patients with complete factors.

Clinical failure and biochemical failure had occurred in 11 and 35 patients who underwent a prostate MRI study, and in 6 and 26 patients without a prostate MRI study, respectively; there were no significant differences between the groups. The patterns of first failure after the 3D-CRT are shown in Table 5; no significant differences were seen between the patients with or without a prostate MRI study. Salvage ADT was performed after a biochemical failure in 35 (19%) patients with a prostate MRI study and 26 (12%) patients without a prostate MRI study. Death from prostate cancer was seen in two (1%) patients who underwent a prostate MRI and in three (1%) patients without a prostate MRI.

**Table 5:** The patterns of first failure after definitive radiotherapy

	Prostate MRI		*p*
	Yes (n = 189)	No (n = 221)	
	No. of patients (%)	No. of patients (%)	
Biochemical failure alone	24 (13)	20 (9)	0.26
Clinical failure	9 (5)	4 (2)	0.10
Primary alone	1	0	
Regional lymph node alone	2	0	
Distant metastasis alone	4	3	
Primary, regional and distant metastasis	1	0	
Regional lymph node and distant metastasis	1	1	

## Discussion

The current study is to assess the benefits, in terms of the tumor staging, CTV and clinical outcomes, of adding prostate MRI examinations to evaluate the T stage in patients with localized prostate cancer treated with 3D-CRT. Although the current study was retrospective and non-randomized in nature, the findings were obtained from a large cohort of patients treated at a single institution. With its excellent soft-tissue resolution, MRI of the prostate clearly depicts the prostate’s zonal anatomy and facilitates prostate cancer localization and staging (Mullerad et al. 2004; Graser et al. 2007; Yu et al. 1997; Engelbrecht et al. 2002). MRI has been shown to contribute significant incremental value to clinical variables in the prediction of the clinical T stage, especially the existence of extracapsular extension and/or seminal vesicle invasion, and to significantly improve treatment planning (Mullerad et al. 2004; Sala et al. 2006; Wang et al. 2004). However, some previous studies also demonstrate that the accuracies of the T staging of prostate cancer between MRI and TRUS were comparable (Rifkin et al. 1990; Presti et al. 1996; Ekici et al. 1999). In the current study, modifications of the CTV for treatment planning with 3D-CRT based on the results of prostate MRI were made in only 8% of the patients. Furthermore, the addition of prostate MRI to TRUS did not improve the bRFS after 3D-CRT in the patients with clinically localized prostate cancer. We therefore confirmed that the clinical benefits of adding prostate MRI to TRUS to determine the T stage are limited in patients with localized prostate cancer treated with 3D-CRT.

As mentioned in the “Background”, the CTV in RT planning for prostate cancer depends on the risk group; for the low risk group, the risk of seminal vesicle involvement is <5%; therefore, the CTV should be restricted to the prostate only. In contrast, for the intermediate to high risk group, the risk of seminal vesicle involvement is higher (over 15%), so the proximal seminal vesicles should be included in the CTV, and for patients with proven seminal vesicle involvement (T3b), the entire seminal vesicle should be included in the CTV (Boehmer et al. 2006; Hayden et al. 2010). In addition, when gross extracapsular disease is recognized under the CTV, a margin of 2–5 mm (excluding the rectum) should be considered (Boehmer et al. 2006; Hayden et al. 2010). In the current study, a modification of the T stage after the prostate MRI was seen in 39 (25%) patients; however, there was a modification of the risk group in only 14 (9%) patients, and eventually, a modification of the CTV in 3D-CRT planning only 13 (8%) patients. Gross extracapsular disease was newly found by the prostate MRI in only six (4%) patients, and all of them had a modification of the CTV in 3D-CRT-planing. Most of the other modifications detected were associated with intracapsular lesions of prostate cancer which did not change the risk group or the CTV. We supposed that these factors may explain why the addition of prostate MRI did not correlate with any improvement of the clinical outcomes.

Prostate MRI adds significantly to the cost of treatment when used routinely. Jager et al. (2000) reported the results of a decision analysis for the appropriate use of MRI for the preoperative staging of prostate cancer, and concluded that MRI staging is cost-effective for patients with a moderate or high prior probability of extracapsular disease. The clinical and pathological parameters predicting extracapsular disease in patients undergoing a radical prostatectomy for clinically localized prostate cancer have been reported in previous studies, and include a smaller prostate volume and positive cores for malignancy from both lobes after prostate biopsy (Sfoungaristos and Perimenis 2012). In the current study, 10 of the 13 patients with a modification of the CTV based on the prostate MRI had gross extracapsular disease. In addition, the modification of the CTV mainly occurred in the patients with high-intermediate risk, high-risk or extreme-risk group on ProCaRS before prostate MRI. Therefore, we also believed that MRI should be performed for select patients with a high probability of extracapsular disease.

Although phased-array MRI on a 1.5T scanner was used in the current study, some previous studies indicated that endorectal coil MRI significantly improved the T staging of prostate cancer (Futterer et al. 2007; Heijmink et al. 2007). Futterer et al. (2007) reported a comparison of the local staging accuracy of pelvic phased-array coil alone versus integrated endorectal-pelvic phased-array coils on a 1.5T scanner, and concluded that the use of endorectal-pelvic phased array coils resulted in significant improvement of extracapsular extension accuracy and specificity. In addition, some studies have reported that the 3.0T phased-array MRI is equivalent to the 1.5T endorectal MRI in evaluating the local staging accuracy for prostate cancer, without a significant loss of imaging quality (Park et al. 2007; Torricelli et al. 2006). Multiparametric MRI, such as anatomic T2-weighted imaging with MR spectroscopic imaging, dynamic contrast-enhanced MR imaging, and diffusion-weighted imaging, also demonstrated great interest in the evaluation of the prostate cancer (Sciarra et al. 2011). Further studies of pelvic MRI using these advanced techniques are therefore needed to evaluate the relationships between the T staging, especially for extracapsular disease, and the clinical benefits.

Our study is associated with several potential limitations. First, due to the fact that the study was retrospective, the possibility of a selection bias with respect to the prognostic factors cannot be ruled out, although we performed both univariate and multivariate analyses of the bRFS. Therefore, in the future, prospective studies will be needed to investigate the actual clinical benefits of prostate MRI. Second, in the current study, modifications of the duration of ADT, based on the results of the addition of prostate MRI, could not be evaluated, because the treatment policy for ADT was variable among the attending urologists, although period of ADT is basically selected depending on the risk group. We believe that the results of prostate MRI would lead to limited or not modification of the period of ADT, because only 14 (9%) patients had a change in risk group after prostate MRI. The duration of ADT in patients who underwent prostate MRI was longer than those in patients who did not, which may have influenced the results of the bRFS, although both the univariate and multivariate analyses demonstrated that the duration of ADT was not a significant factor on the bDFS. Third, hyperthermia was added to 3D-CRT to obtain radio-sensitization in select patients due to the lower total dose of 3D-CRT, which may have led to a bias in the study. Several phase II clinical trials on radiotherapy combined with hyperthermia showed promising results in patients with prostate cancer (Maluta et al. 2007; Anscher et al. 1992; Van Vulpen et al. 2004). However, in the current study, the addition of hyperthermia was not a significant factor on the bRFS. Fourth, patients who received early treatment were more likely to have undergone prostate MRI. Therefore, the follow-up duration in the patients who underwent prostate MRI was longer than that in the patients who did not. The difference in the follow-up duration may have had an impact on the results of the bRFS, although the date of the treatment was not a significant factor in the univariate/multivariate analyses on the bRFS (Table 4).

In summary, modifications of the CTV for treatment planning with 3D-CRT based on the results of prostate MRI were identified in only 8% of the patients in the present study, most of whom had extracapsular disease. In contrast, modification of the T stage after prostate MRI was observed in approximately one-quarter of the patients, and the addition of prostate MRI was not associated with any improvements in outcomes after 3D-CRT. Therefore, the clinical benefits of adding prostate MRI to evaluate the T stage may be limited in patients with localized prostate cancer treated with 3D-CRT. Prostate MRI should not be performed in every patient with clinically localized prostate carcinoma, but is recommended for the patients with a high prior probability of extracapsular disease.
